# Standard of care drugs do not modulate activity of senescent primary human lung fibroblasts

**DOI:** 10.1038/s41598-023-30844-0

**Published:** 2023-03-04

**Authors:** Stephanie Badaro-Garcia, Miriam S. Hohmann, Ana Lucia Coelho, Waldiceu A. Verri, Cory M. Hogaboam

**Affiliations:** 1 Women’s Guild Lung Institute, Cedars-Sinai Medical Center, Los Angeles, USA; 2 Laboratory of Pain, Inflammation, Neuropathy, and Cancer, Department of Pathology, Londrina State University, Londrina, Brazil

**Keywords:** Senescence, Pathogenesis

## Abstract

Cellular senescence is crucial in the progression of idiopathic pulmonary fibrosis (IPF), but it is not evident whether the standard-of-care (SOC) drugs, nintedanib and pirfenidone, have senolytic properties. To address this question, we performed colorimetric and fluorimetric assays, qRT-PCR, and western blotting to evaluate the effect of SOC drugs and D + Q on senescent normal and IPF lung fibroblasts. In this study, we found that SOC drugs did not provoke apoptosis in the absence of death ligand in normal or IPF senescent lung fibroblasts. Nintedanib increased caspase-3 activity in the presence of Fas Ligand in normal but not in IPF senescent fibroblasts. Conversely, nintedanib enhanced B cell lymphoma 2 expression in senescent IPF lung fibroblasts. Moreover, in senescent IPF cells, pirfenidone induced mixed lineage kinase domain-like pseudokinase phosphorylation, provoking necroptosis. Furthermore, pirfenidone increased transcript levels of *FN1* and *COL1A1* in senescent IPF fibroblasts. Lastly, D + Q augmented growth differentiation factor 15 (GDF15) transcript and protein levels in both normal and IPF senescent fibroblasts. Taken together, these results establish that SOC drugs failed to trigger apoptosis in senescent primary human lung fibroblasts, possibly due to enhanced Bcl-2 levels by nintedanib and the activation of the necroptosis pathway by pirfenidone. Together, these data revealed the inefficacy of SOC drugs to target senescent cells in IPF.

## Introduction

Idiopathic pulmonary fibrosis (IPF) is chronic and progressive idiopathic interstitial lung disease, which is characterized by histopathologic and/or radiologic findings of usual interstitial pneumonia^1,2^. IPF is an age-related disease that commonly manifests in individuals older than 50 years, and its median survival is approximately 3 to 5 years after diagnosis^2^. The disease course is heterogeneous and includes dyspnea, worsening lung function, and impaired quality of life^3,4^.

Several risk factors have been described to enhance the risk of IPF, including genetics, gender, aging, comorbidities, smoking, environmental and occupation exposure. Nonetheless, aging is the most prominent factor, and recent studies have highlighted the contribution of senescence cells in IPF^5^. Cellular senescence is one of the hallmarks of aging, characterized by cell cycle arrest and secretion of an array of chemokines, pro-inflammatory cytokines, growth factors, and extracellular matrix proteases, thus comprising a secretome referred to as the senescence-associated secretory phenotype (SASP)^6^. Although many cellular senescence hallmarks have been described, the senescence phenotype is diverse, and its mechanisms are not conserved among various cell types^7^. Accumulating evidence demonstrates that senescent cells play a deleterious role in IPF, and it has been shown that the removal of senescent cells increases the life span in animal models^8^. However, the way senescent cells exacerbate the disease remains unclear.

Lung fibroblasts are essential in wound healing in response to lung injury^9^. Upon lung epithelium damage, activated fibroblasts differentiate into myofibroblasts and migrate to the injury site, producing extracellular matrix (ECM) components promoting tissue repair^10^. On the one hand, accumulated fibroblasts become senescent and reduce their ECM deposition, limiting the fibrotic process. On the other hand, evidence has shown that targeting senescent fibroblasts reduces pulmonary fibrosis in mice models. That stated, the identification of mechanisms to remove senescent cells would have a remarkable impact on the quality of life and burden of IPF.

Nintedanib (Ofev, Boehringer Ingelheim) and pirfenidone (Esbriet, InterMune) are two drugs approved for the treatment of IPF^11^. Nintedanib, a tyrosine kinase inhibitor, blocks the effects of platelet-derived growth factor, fibroblast growth factor, and vascular endothelial growth factor receptor and inhibits signaling pathways in the proliferation, migration, and differentiation of lung fibroblasts^12,13^. Whereas pirfenidone (whose precise mechanism of action remains unclear) exerts anti-fibrotic, antioxidant, and anti-inflammatory effects to reduce lung collagen synthesis and deposition in bleomycin animal models. Although these standard-of-care (SOC) drugs are anti-fibrotic neither drug truly modify disease nor significantly improve the quality of life of IPF patients^14^. Senolytics are drugs that can selectively induce senescent cells apoptosis^15^. Dasatinib plus Quercetin (D + Q), a tyrosine kinase inhibitor and a flavonoid with antioxidant properties, respectively, constitutes the first combination of senolytics described^16^. D + Q ameliorated bleomycin-induced pulmonary fibrosis and improved pulmonary and physical function^16^.

Taken together, the relevance of senescence in IPF and the fact SOC drugs are being broadly used, herein we evaluate whether SOC drugs exert senolytic or senomorphic effects and compare them to D + Q on apoptosis-resistant senescent primary human lung fibroblasts and we evaluated the types of cell death type induced after SOC drugs treatment of these lung cells.

## Results

### Lung fibroblasts isolated from normal and IPF patients exhibited a senescent phenotype after underwent replicative senescent

**Figure 1:**
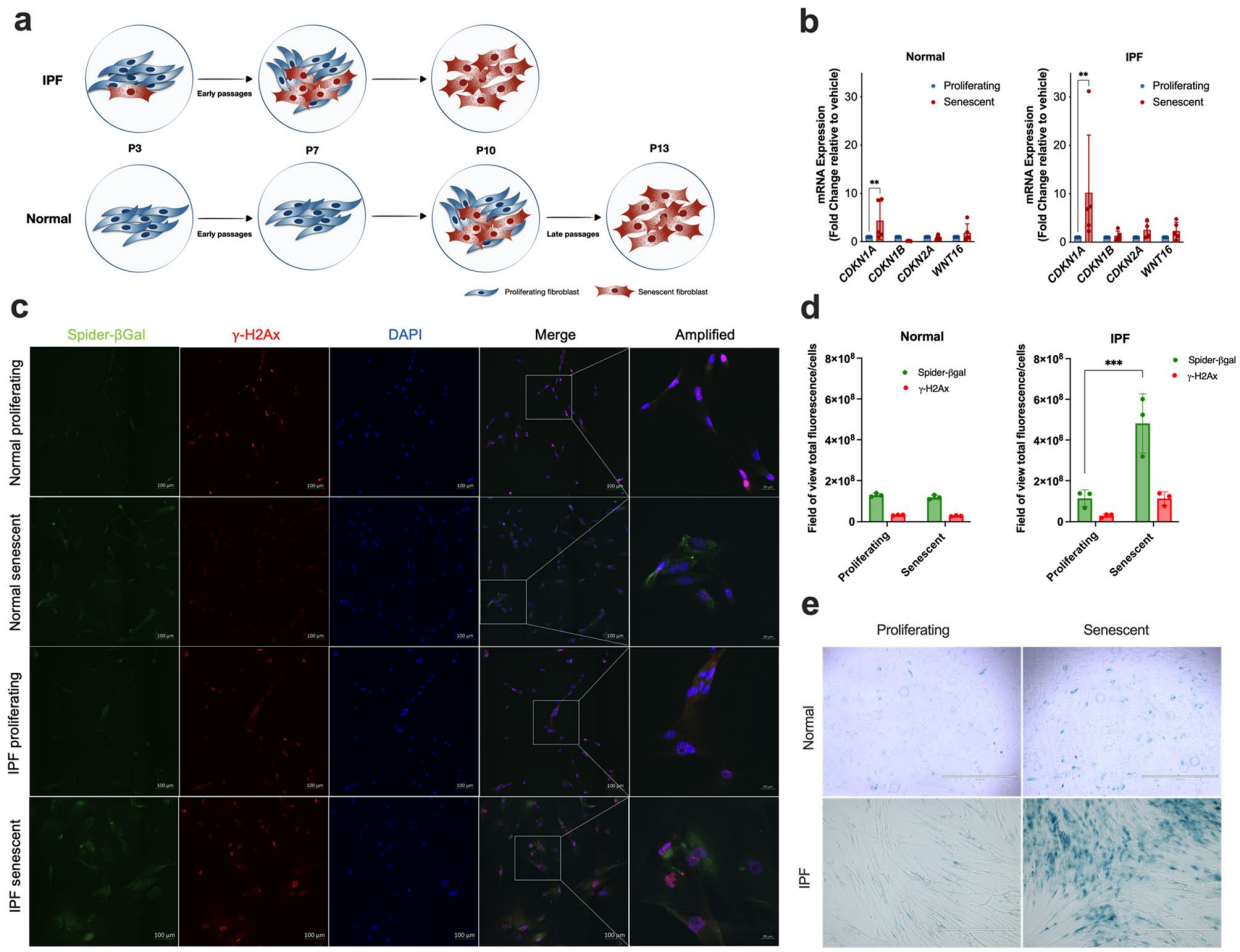
Lung fibroblasts isolated from normal and IPF patients exhibited a senescent phenotype after underwent through replicative senescence. Schematic presentation of normal and IPF lung fibroblasts transition from early to late passages (**a**). mRNA expression of senescence associated genes *CDKN1A*, *CDKN1B*, *CDKN2A*, and *WNT16* in proliferating and senescent lung fibroblasts from normal or IPF patients (**b**). Representative images of SA-β-gal and the DNA damage marker γ-H2Ax co-staining is pronounced in senescent lung fibroblasts from normal or IPF patients (**c**). The scale bars indicate 100 μM (Spider-β-gal, γ-H2Ax, DAPI and merge) and 20 μM for amplified image. Quantification of SA-β-gal and γ-H2Ax total fluorescence/number of cells from proliferating and senescent lung fibroblasts from normal or IPF patients (**d**). SA-β-galactosidase staining is detected in senescent lung fibroblasts from normal and IPF patients but not in proliferating lung fibroblasts (**e**). Data are presented as mean ± SD (n = 3 or 5 per group). *p < 0.05, **p < 0.01 and ***p < 0.001 as indicated by the bars.

First, we performed series of continual passaging of proliferating normal and IPF lung fibroblasts to induce replicative senescence (Fig. 1a). To confirm the status of the lung fibroblasts before we proceed with the next experiments, we performed co-staining of SA-β-gal and the DNA damage marker γ-H2Ax, a SA-β-Gal staining and a RT-PCR for the senescence markers *CDKN1A*, *CDKN1B*, *CDKN2A* and *WNT16.* The elevated expression of p21 (CDKN1A), an important marker of cellular senescence, was observed in senescent when compared to proliferating lung fibroblast in both normal and IPF cells (Fig. 1b). Additionally, we observed that senescent lung fibroblasts exhibited increased SA-β-gal and γ-H2Ax fluorescence compared to proliferating lung fibroblasts (Fig. 1c, d). In addition, SA-β-Gal staining also confirmed the abundance of SA-β-Gal in senescent cells but not in proliferating lung fibroblasts (Fig. 1e). Moreover, we detected the phenotypic difference between proliferating (elongated and spindle-shaped) and senescent lung fibroblasts (enlarged and irregularly shaped). Together these data confirmed the status of normal and IPF fibroblasts as senescent, which allowed that we proceed to the next experiments.

### SOC drugs do not alter cell viability, LDH leakage, or caspase-3 release

**Figure 2:**
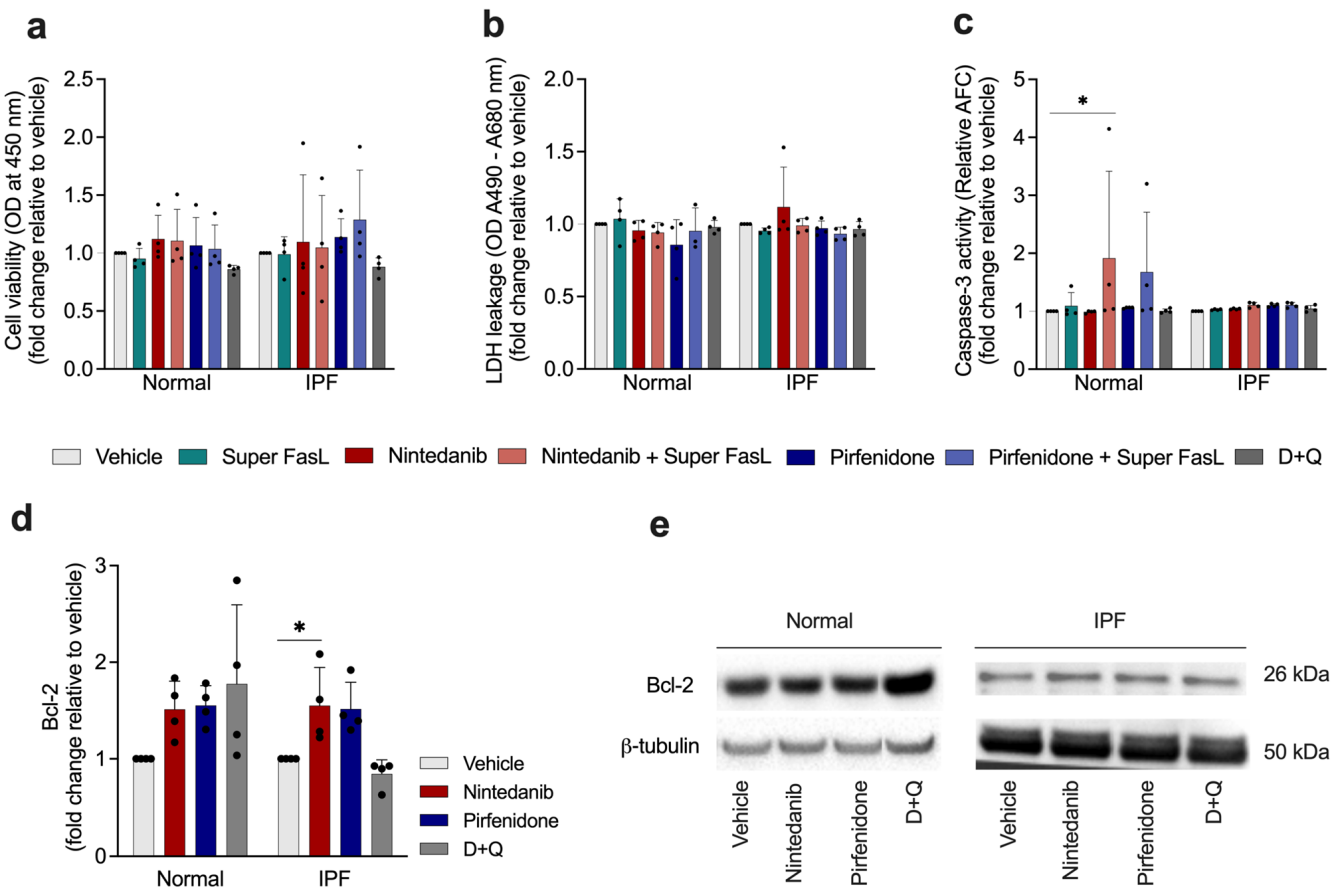
SOC drugs did not trigger apoptosis while nintedanib enhances b-cell lymphoma 2 (Bcl-2) protein levels. Cell viability, lactate dehydrogenase (LDH) release and caspase-3 activity by senescent lung fibroblasts from normal and IPF patients after 24-h treatment with vehicle (DMSO 0.05%), nintedanib (300 nM), pirfenidone (2.5 mM), or D + Q (20 μM/15 μM) followed by 3 h of Super Fas Ligand (Super FasL, 100 ng/ml) (**a–c**). Western blot quantification of Bcl-2 protein levels (**d**) in senescent normal and IPF fibroblasts lysates. Representative western blot of Bcl-2 protein levels (**e**). Data are presented as mean ± SD (n = 3 or 4 per group). *p values were calculated using one-way ANOVA followed by Tukey’s test.* *p < 0.05 as indicated by the bars.

Next, we explored the hypothesis that SOC drugs modulate apoptosis in senescent normal and IPF lung fibroblasts in the presence or absence of a proapoptotic stimuli. We treated these cells with vehicle (DMSO) 0.05%, nintedanib (300 nM), pirfenidone (2.5 mM), or D + Q (20 μM /15 μM) for 24 h, followed by the incubation with 100 ng/mL recombinant FasL protein (Super FasL). Neither nintedanib nor pirfenidone showed an effect on cell viability (Fig. 2a) or LDH leakage (Fig. 2b), on normal or IPF senescent lung fibroblasts. However, nintedanib increased caspase-3 activity when combined with the cell death ligand Fas only on normal cells (Fig. 2c). Given these findings, we next sought to evaluate the influence of SOC drugs and D + Q on the apoptosis regulator Bcl-2. We saw an increase in Bcl-2 protein levels after the treatment with nintedanib (Fig. 2d-e), and the same effect was not observed in cells treated with pirfenidone or D + Q. Collectively, these data demonstrate that SOC drugs do not trigger apoptosis in senescent lung fibroblasts.

### Expression of marker of senescence in normal and IPF senescent fibroblasts after treatment with standard of care drugs and D + Q

**Figure 3:**
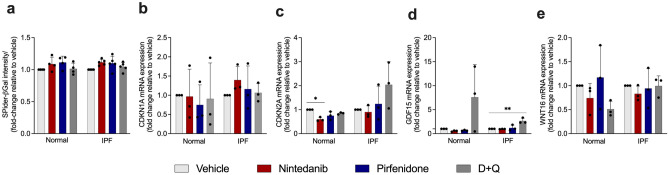
SOC drugs do not alter the senescent phenotype of senescence lung fibroblasts. Senescent fibroblasts isolated from the lungs of normal patients or IPF patients were plated simultaneously at time 0 and treated with vehicle (DMSO 0.05%) nintedanib (300 nM), pirfenidone (2.5 mM), or D + Q (20 μM/15 μM) for 24 h. Beta-galactosidase intensity (**a**), *CDKN1A* (**b**), *CDKN2A* (**c**), *GDF15* (**d**)*,* and *WNT16* (**e**) transcripts were first normalized to the housekeeping gene *18S*. Data are presented as mean ± SD (n = 3–5 per group). *p < 0.05, **p < 0.001 as indicated by the bars.

Normal and IPF lung fibroblasts were cultured through serial passage rounds until cells obtained the senescence phenotype, as previously described^17^. One of the most used methods to evaluate cellular senescence is the detection of β-Gal activity^18^. We assessed the influence of SOC drugs on senescent cells by measuring Spider-βGal intensity, however, we did not find any significant difference after the treatment with SOC drugs or D + Q (Fig. 3a). We next determined the expression of *CDKN1A* (p21) and *CDKN2A* (p16) used as markers of cellular senescence^19^. We observed a reduction of *CDKN2A* mRNA expression only in normal lung fibroblasts after the treatment with nintedanib (Fig. 3b, c). In addition, we evaluated GDF15 gene expression, which it has been described as one of the components of SASP^20^. Interestingly, we observed that *GDF15* was upregulated after D + Q treatment in IPF fibroblasts, which was not observed in any of the other treatments (Fig. 3d). Moreover, Wnt16 emerged as a new marker of senescence, regulating p53 activity and phosphatidylinositol 3-kinase (PI3K)/Ak Strain Transforming (AKT) pathway^21^. We did not observe any significant difference in *WNT16* mRNA expression among the treated groups (Fig. 3e).

### Influence of SOC and D + Q on senescent lung fibroblasts

**Figure 4:**
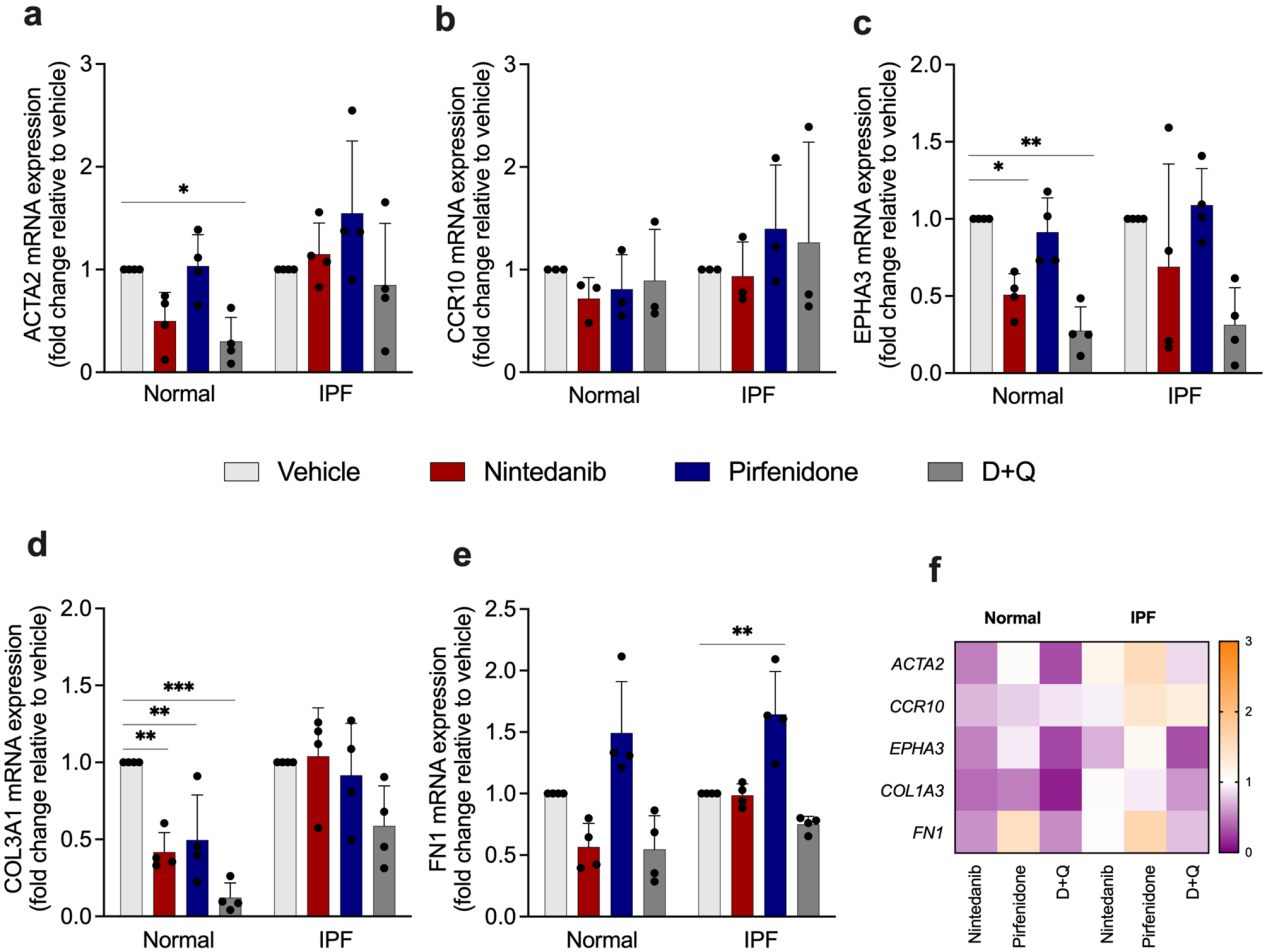
Effects of SOC drugs and D + Q on *ACTA2*, *CCR10*, *EPHA3, COL3A1*, and *FN1* mRNA expression in normal and IPF senescent lung fibroblasts. Heatmap of the expression of SASP and fibrosis-related genes in lung fibroblasts from normal and IPF patients treated with nintedanib (300 nM), pirfenidone (2.5 mM), D + Q (20 μM/15 μM) for 24 h. *ACTA2* (**a**), *CCR10* (**b**), *EPHA3* (**c**)*, COL3A1* (**d**) and *FN1* (**e**) transcripts were first normalized to the housekeeping gene *18S* Each transcript was first normalized to the housekeeping gene RNA *18S*. Upregulation (orange) and downregulation (purple) of gene expression, compared with vehicle-treated cells (**f**). Data are presented as mean ± SD (n = 3 or 4 per group). p values were calculated using one-way ANOVA followed by Tukey’s test. *p < 0.05; **p < 0.001; and ***p < 0.0001 as indicated by the bars.

We next examined whether SOC drugs and D + Q influenced the expression of *ACTA2*, *CCR10*, *EPHA3, COL3A1*, and *FN1*. The treatment with D + Q was able to reduce *ACTA2* mRNA expression only in normal senescent lung fibroblast, while the other drugs did not show an effect on *ACTA2* expression (Fig. 4a). We did not identify a significant change in *CCR10* mRNA expression after the treatment (Fig. 4b). We observed a reduced *EPHA3*, a mesenchymal marker, after the treatment with nintedanib and D + Q in normal senescent lung fibroblasts, while the same effect was not observed in IPF cells with any treatment (Fig. 4c). Moreover, all drugs reduced *COL3A1* mRNA expression in normal senescent lung fibroblast. However, the same effect was not observed in IPF cells (Fig. 4d). The deposition of extracellular matrix proteins, like collagen and fibronectin in the lung, triggers respiratory failure^22^. Surprisingly, the treatment with pirfenidone enhanced *FN1* in IPF senescent lung fibroblast (Fig. 4e). The heatmap shows the upregulation (orange) and downregulation (purple) of SASP and fibrosis-related genes (Fig. 4f).

### Pirfenidone increases COL1A1 expression in senescent IPF fibroblasts, and D + Q reduces collagen expression and release in senescent normal and IPF fibroblasts

**Figure 5:**
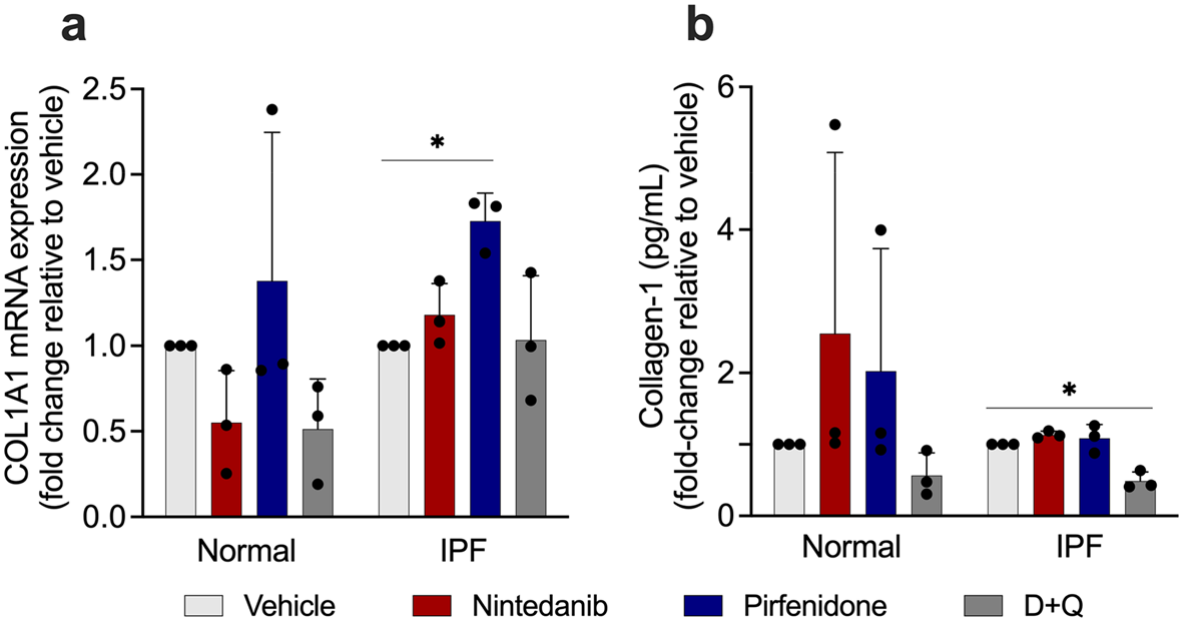
Influence of SOC drugs on *COL1A1* mRNA expression and type I collagen secretion. Effects of nintedanib (300 nM), pirfenidone (2.5 mM), D + Q (20 μM/15 μM) or vehicle (DMSO 0.05%), on *COL1A1* mRNA expression (**a**) and collagen-1 levels (**b**). *COL1A1* transcript was first normalized to the housekeeping gene RNA *18S*. Data represented as mean ± SD (n = 3 per group). p values were calculated using one-way ANOVA followed by Tukey’s test. *p < 0.05. **p < 0.01 as indicated by the bars.

One of the fibrosis hallmarks is the excessive deposition of fibrotic extracellular matrix proteins, especially type I collagen^23^. To determine the effects of SOC drugs on the fibrosis-related marker type I collagen, we investigated the expression of *COL1A1* in senescent lung fibroblasts after the treatment with nintedanib, pirfenidone, or D + Q. RT-PCR and ELISA demonstrated that the expression levels of *COL1A1* were significantly increased in the IPF group after pirfenidone treatment (Fig. 5a). On the other hand, D + Q treatment significantly decreased type I collagen secretion in normal and IPF lung fibroblasts (Fig. 5b).

### SOC drugs influence IL-6, IL-8, MCP-1, GDF-15, Wnt16 in senescent cells

**Figure 6:**
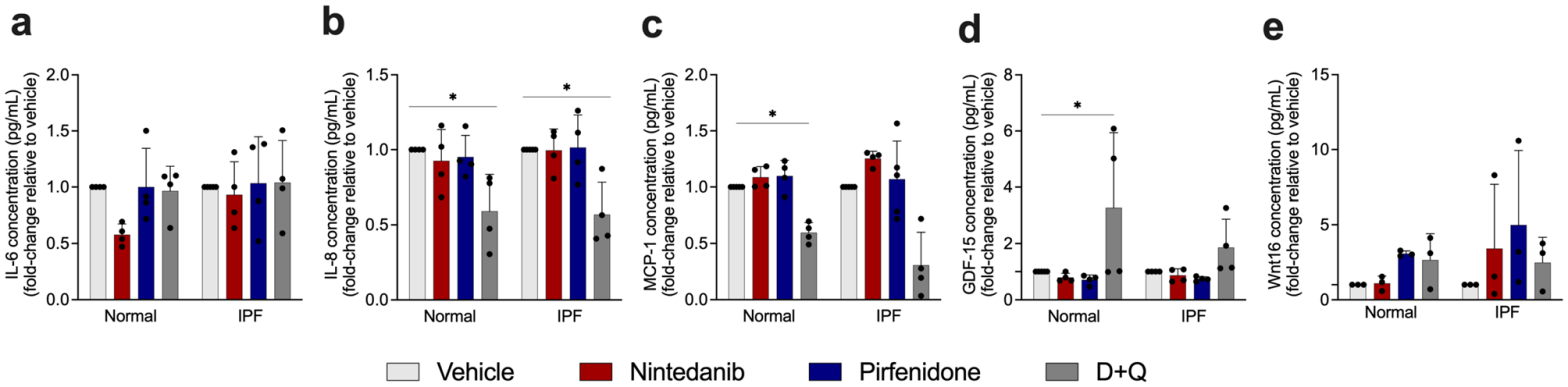
SOC do not have an impact on SASP release. Effects of treatment for 24 with nintedanib (300 nM), pirfenidone (2.5 mM), D + Q (20 μM/15 μM) or control (vehicle; DMSO 0.05%), on IL-6 (**a**), IL-8 (**b**), MCP-1 (**c**), GDF-15 (**d**), and Wnt16 (**e**) levels in the supernatant of normal and IPF senescent lung fibroblasts. Data are presented as mean ± SD (n = 3 or 4 per group). p values were calculated using one-way ANOVA followed by Tukey’s test. p* < 0.05 and **p < 0.001 as indicated by the bars.

Next, we investigated whether SOC drugs affect senescent cells via modulation of their secretome. Senescent cells secrete interleukins, inflammatory cytokines, and growth factors that can affect neighboring cells^24^. Among SASP cytokines, the pleiotropic pro-inflammatory cytokine IL-6 is the most distinguished. We observed that SOC drugs and D + Q did not affect IL-6 in both cell types (Fig. 6a). The treatment with D + Q reduced IL-8, a pro-inflammatory cytokine found in SASP, in senescent normal and IPF lung fibroblasts (Fig. 6b). Taking into consideration that senescent fibroblast can recruit leukocytes, and MCP-1 is a crucial component of SASP^25^, we evaluated the concentration of MCP-1 on normal and IPF senescent fibroblasts. The treatment with D + Q was able to reduce MCP-1 release in normal but not in IPF fibroblast, while SOC drugs showed no effect neither in normal nor IPF senescent fibroblast (Fig. 6c). In addition, compelling studies showed GDF-15 emerging as part of the SASP repertoire^26^. After the dosage of GDF-15, we observed a prominent increase after D + Q treatment only in normal senescent fibroblasts. However, no significant results were observed after the treatment with SOC drugs in normal or IPF senescent cells (Fig. 6d). Moreover, we observed that after the treatment with SOC drugs and D + Q, there was no significant difference in Wnt16 release when compared with vehicle, showing that those drugs do not affect Wnt16 (Fig. 6e).

### Pirfenidone triggers necroptosis in IPF lung fibroblasts

**Figure 7:**
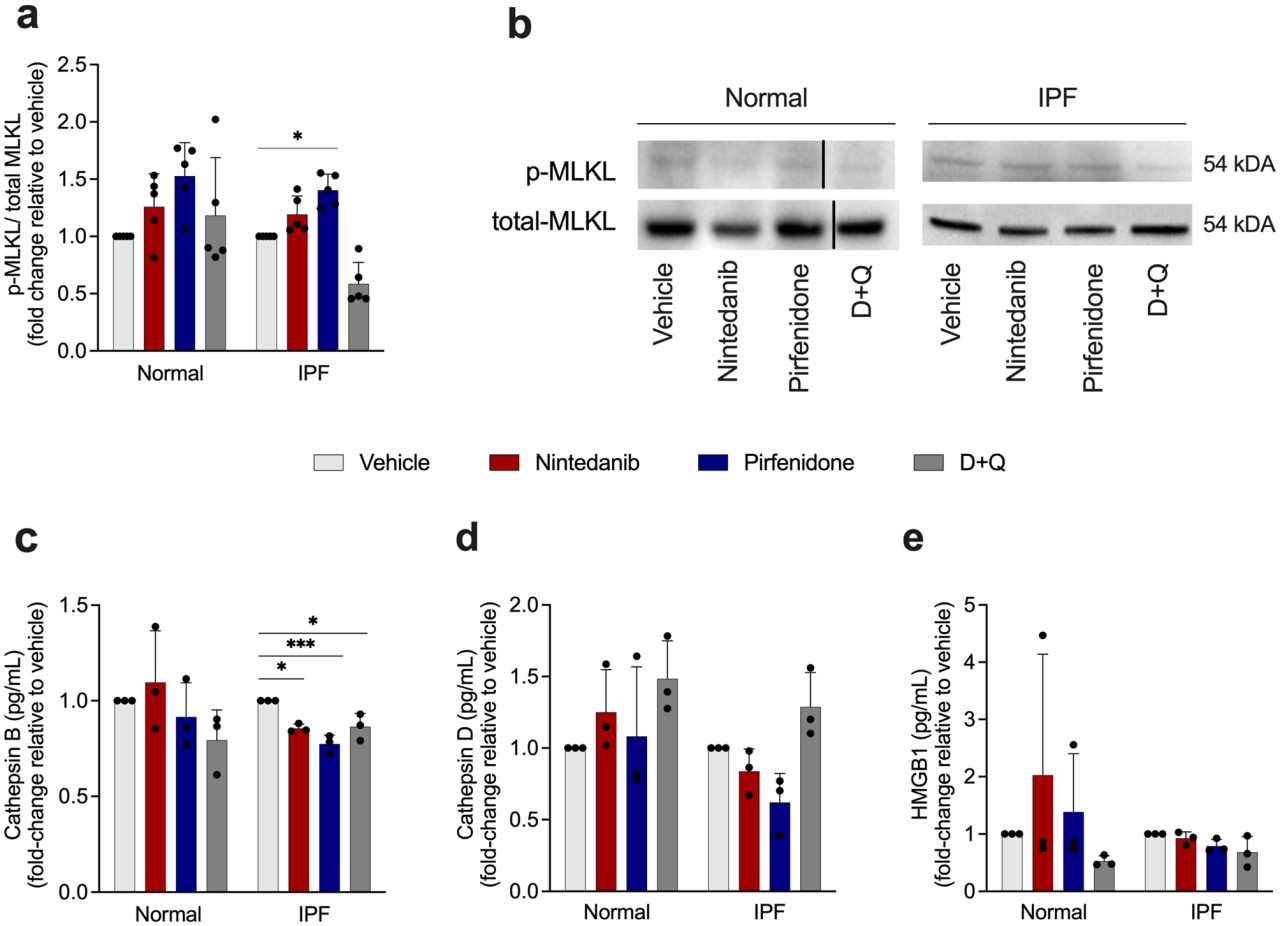
Pirfenidone enhances MLKL phosphorylation and reduces cathepsin B release. Western blot quantification of MLKL phosphorylated/MLKL total of normal and IPF senescent lung fibroblasts treated for 24 h with nintedanib (300 nM), pirfenidone (2.5 mM), D + Q (20 μM/15 μM) or control (vehicle; DMSO 0.05%) (**a**). Representative western blot of p-MLKL/MLKL protein expression (**b**). Cathepsin B (**c**), cathepsin D (**d**) and HMGB1 (**e**) levels in senescent fibroblasts supernatant after treatment with nintedanib (300 nM), pirfenidone (2.5 mM), D + Q (20 μM/15 μM) or control (vehicle; DMSO 0.05%) for 24 h. Data are presented as mean ± SD (n = 3 or 5 per group). p values were calculated using one-way ANOVA followed by Tukey’s test. *p < 0.05 **p < 0.01 and ***p < 0.001 as indicated by the bars.

Subsequently, to address whether SOC drugs trigger other cell death types, we performed western blot to measure phosphorylated MLKL (p-MLKL) and total MLKL in senescent fibroblasts from normal and IPF patients. The activation of MLKL upon its phosphorylation initiates necroptosis, a form of programmed cell death in which rupture of cellular membranes leads to the release of intracellular components^27^. Treatment with pirfenidone significantly increases the ratio p-MLKL/MLKL (Fig. 7a, b), concluding that this drug leads to necroptosis. Although Nintedanib presented a mild increase of p-MLKL/MLKL, it was not significant when compared to the vehicle. We next evaluated the role of SOC drugs in necroptosis secreted factors, the release of cathepsin B, cathepsin D, and HMGB1. We observed that the treatment with nintedanib, pirfenidone and D + Q were able to reduce the levels of cathepsin B in IPF senescent lung fibroblast (Fig. 7c), while no significant difference was observed in the normal group. Moreover, any of the treatments influenced cathepsin D and HMGB1 release (Fig. 7d, e).

## Discussion

Senescence is a well-established feature in IPF^28^. Senescent cells dictate the generation of several age-related phenotypes, and their removal from aged tissues can prevent or delay organ dysfunction and lengthen healthspan^29^. Notwithstanding, senescent cells are remarkably resistant to apoptosis due to the upregulation of several proteins that contribute to anti-apoptotic pathways^30^, the clearance of senescence cells has been shown to protect mice from lung fibrosis^31^. Under this senescent state, the cells secrete a myriad of cytokines and proteases and growth factors that play a pivotal impact on adjacent cells and the tissue microenvironment^32^.

In the present study, we demonstrate that the treatment with IPF SOC drugs did not evoke apoptosis in senescent IPF fibroblasts, either spontaneously or via pro-apoptotic ligand FasL. This resistance to apoptosis is consistent with the findings of Moodley et al. who showed that myofibroblasts isolated from fibrotic lungs were more resistant to Fas ligand-induced apoptosis than control myofibroblasts when examined *in vitro*^33^. The cocktail of D + Q has shown remarkable results in reducing pulmonary fibrosis in a bleomycin-induced fibrosis model, and this drug combination also drives cell death in radiation-induced senescent fibroblasts^16^. Surprisingly, our study also shows that D + Q fails to trigger cell death in senescent IPF lung fibroblasts, and these results might reflect the time point examined after treatment (i.e., 24 h) or our method used for inducing cellular senescence in primary human lung fibroblasts (i.e., replicative senescence versus stress-induced or oncogene driven senescence). Moreover, the accumulation of proteins from the Bcl-2 family contributes to the resistance to undergoing apoptosis^34^. Here, we discovered that, senescent IPF cells treated with nintedanib presented elevated levels of Bcl-2 when compared to normal senescent cells, despite previous studies showing nintedanib inhibits apoptotic proteins in lung-resident myofibroblasts^35^, although the effect was not evaluated in senescent cells, which are naturally resistant to apoptosis^36^. Although Cho et al. (2022) demonstrate that nintedanib triggers intrinsic apoptosis in human dermal senescent fibroblasts by inhibiting STAT3 pathway, we believe this discrepancy is due the higher concentration that was used their studies (2–20 μM)^37^ in contrast to 300 nM concentration for 24 h that we used in our studies, using senescent lung fibroblasts from patients and donors.

Mounting evidence indicates the progression of fibrosis involves ECM-driven mechanisms. Mesenchymal cells and their ECM products lead to the expansion of the alveolar wall, causing loss of the gas-exchange surface. The appearance of myofibroblast foci in IPF coincides with the production of several ECM components such as type I and III collagens, extra domain fibronectin, and fibrin, all of which contribute to progression in IPF^38^. The treatment with nintedanib diminished EPHA3 and COL3A1 expression in normal senescent fibroblast but no significant effect was found in IPF cells. We observed an increase in fibronectin 1 (*FN1*) and collagen 1 (*COL1A1*) gene expression after the treatment with pirfenidone, suggesting that although evidence shows its anti-fibrotic effect in proliferative IPF fibroblasts^39^, our results suggest that pirfenidone was unable to reduce those fibrosis markers. In agreement with our findings, Roach et al. (2021) and collaborators found increased deposition of collagen 1 and secretion of soluble collagen after treating fibroblasts with pirfenidone^40^. Importantly, in the present study, we show that the cocktail D + Q reduced ACTA2, EPHA3 and COL3A1 expression in normal senescent fibroblasts, but this effect was not observed in IPF senescent fibroblasts. However, we found a decrease of soluble collagen 1 in senescent IPF fibroblasts.

In a bleomycin model, GDF-15 was previously observed to be the most upregulated protein and this cytokine appears to be a suitable biomarker of epithelial stress and severity of fibrotic lung disease^20^. Moreover, Tanaka et al. used a proteomics analysis of lung samples and observed a positive association between GDF15 and age^26^. In addition, GDF15 is reported to increase α-SMA expression in WI-38 lung fibroblasts, suggesting that elevated GDF15 in the fibrotic lung contributes to the fibrotic process^41^. Intriguingly, after D + Q treatment of senescent IPF fibroblasts, GDF-15 levels were significantly higher when compared to SOC drugs, suggesting that D + Q might be altering the synthetic activity of senescent IPF, which in turn might further contribute to the fibrotic process mediated by fibroblast progenitor or progeny.

Since pro-apoptotic caspase-3 release was not detected after SOC treatment in senescent IPF cells, we investigated whether other forms of cell death might be activated via these drugs. Necroptosis is an alternative form of cell death when caspase-dependent apoptosis is restricted or absent^42^. It is present in many pathologies such as inflammatory diseases^43^, ischemia–reperfusion injuries^44^, and degenerative diseases^45^. It has been demonstrated that augmented levels of Receptor Interacting Serine/Threonine Kinase 3 (RIPK3) and phosphorylated MLKL in alveolar epithelial cells (AECs) in IPF lungs^46^ leading Lee et al. to postulate that cell-damaging agents injure AECs, leading RIPK-3–regulated necroptosis. In addition, damage-associated molecular patterns (DAMPS), including HMGB1 and IL-β, released from necroptotic AECs are responsible not only for inflammation but also for the development of fibrosis through enhanced myofibroblast differentiation during IPF pathogenesis. The necroptosis pathway can be induced by impaired apoptosis by ligand-dependent stimulation of cell death receptors, for instance, Fas^47^. It is orchestrated by distinct proteins, namely RIPK1 and RIPK3 and the downstream protein MLKL, that once phosphorylated by RIPK3, lead to necroptosis by inducing the formation of oligomers, migration to the plasma membrane, and binding to phosphatidylinositol lipids to directly disrupt membrane integrity^48^. In the present study, we found some evidence that pirfenidone increases MLKL phosphorylation, the major hallmark of necroptosis, and the same effect was not observed after the treatment with nintedanib or D + Q. However, the mechanism whereby pirfenidone enhances this phosphorylation requires further investigation.

Previous work has shown that the inhibition of cathepsins provokes the induction of necroptosis in bone marrow-derived macrophages, suggesting that cathepsins act as anti-necroptotic factors^49^. This finding corroborates with our findings in the present study since we observed a decrease in cathepsin B after the treatment with nintedanib, pirfenidone, and, interestingly, D + Q. Similar results were obtained with cathepsin D. Moreover, cathepsins are involved in the degradation of the anti-apoptotic protein Bcl-2, leading to apoptosis^50^. However, SOC drugs diminished cathepsin activity, which could potentially trigger cellular evasion of apoptosis and thereby increase the phosphorylation of MLKL ultimately leading to necroptosis.

We would like to acknowledge limitations in this study such as striking differences observed among the same group in IPF. We strongly believe that this phenomenon is due differences between the two fibroblasts subsets, brilliantly described by Levesque et al*.* (2021)^51^.

Although SOC have been reported to be an important clinical strategy to treat IPF patients, over the years, the advance of senotherapeutics in preclinical model aligned with outstanding preliminary results of clinical trials, have proved the importance of targeting senescent cells in IPF, and clearly, SOC did not have a significant impact on senescent lung fibroblasts .

Together, these data demonstrate that SOC drugs fail to promote apoptosis or modulate the synthetic capacity of senescent IPF fibroblasts. Moreover, pirfenidone appeared to promote inflammatory cell death, however the mechanism which this drug mediates necroptosis requires future investigation. Indeed, further studies are necessary to determine whether SOC drugs prevent the appearance of senescent cells such as fibroblasts in IPF. Overall, this study sheds light on the need to develop more effective therapies for IPF, which target and selectively eliminate senescent cells in this progressive fibrotic lung disease.

## Methods

### Senescent fibroblast generation

Primary normal lung fibroblasts were derived from nonfibrotic lung samples without signs of disease from lung biopsies and primary IPF lung fibroblasts were derived from IPF patients from lung explants (Table 1). To obtain senescent fibroblasts, proliferating normal and IPF lung fibroblasts were repeatedly passaged in culture until they reached a senescent morphological phenotype (enlarged, flattened, and irregular shape) and SA-β-gal activity^17^. Normal lung fibroblasts reached cellular senescence within 12–15 passages and IPF lung fibroblasts became senescent after 9–11 passages. Senescent fibroblasts were cultured in Dulbecco's Modified Eagle Medium (DMEM; Lonza, Basel, Switzerland) supplemented with 15% FBS (Atlas Biologicals, Inc, Fort Collins, CO), 1% penicillin/streptomycin (Mediatech, Manassas, VA), 1% glutamine (Mediatech) and 0.1% of primocin (Invivogen, San Diego, CA) at 37 °C, and 10% CO_2_.

**Table 1:** IPF patient demographics.

Sample name	Age	Gender	Diagnosis	Smoking history
CCA-18	67	Male	Normal	Smoked 1–2 cigarettes primarily socially at age 18
CC01-19	47	Male	Normal	Smoked 0.5–1.5 packs of cigarettes for 35 years. Did not quit smoking
CC07-19	18	Male	Normal	Nonsmoker
CC02-20	62	Male	Normal	Smoked 3 Cigarettes/day for 27 years. Quit at age 42
CC04-20	60	Male	Normal	Smoked less than 1 pack per week from 1978–1980. Quit in 1980
CC06-20	57	Male	Normal	Cigarettes, ½ pack since age 16
CC01-21	54	Male	Normal	No history but lungs showed signs of smoking
IPF10-18	66	Male	IPF	Nonsmoker
IPF14-19	58	Male	IPF	Former smoker. Quit at 09/03/1994
IPF01-20	68	Male	IPF	Nonsmoker
IPF03-20	66	Female	IPF	Nonsmoker
IPF04-20	69	Male	IPF	Nonsmoker
IPF05-20	73	Male	IPF	Former smoker. Smoked 2.5 packs/day for 25 years. Quit 21.3 years ago
IPF08-20	64	Male	IPF	Never smoker
IPF07-21	74	Female	IPF	Nonsmoker

### Co-staining of SA-β-gal and the DNA damage marker γ-H2Ax

Proliferating and senescence cells were plated in an 8-chamber slide (Thermo Scientific, Waltham, MA, USA) at 37 °C overnight in a 5% incubator. SA-β-gal staining was performed following manufacturer’s protocol for living cells (Dojindo Cat #SG03-10). Next, cells were fixed in 4% paraformaldehyde for 15 min at room temperature (RT). After three washes, cells were permeabilized with 0.1% Triton X-100/PBS for 30 min and blocked in 1% BSA/PBS for 1 h. An anti- *γ-*H2A.x antibody (Cell Signaling Cat#2577L) diluted in 1% BSA/PBS was added to the cells and incubated overnight at 4 °C. After 3 washes, an anti-rabbit secondary antibody (ThermoFisher Scientific, Alexa Fluor 594 Cat#A11012) was added to the cells and incubated for 2 h at RT. Cells were washed with PBS 3 times and incubated with 2 μg/mL DAPI (ThermoFisher Scientific, Cat#62,248) for 10 min. The same washing process was repeated, and cells were observed under a confocal microscope and analyzed using Zen 2.5 blue edition software and Image-Pro Premier E9.2 software.

### SA-β-Gal staining

SA-β-Gal staining was performed following manufacturer’s protocol (BioVision Inc., Milpitas, CA, US; Cat #K320-250). Cells were observed under light microscopy for the development of blue color.

### Senescence associated β-galactosidase detection

To assess SA-β-galactosidase levels, a cellular senescence assay was performed (Dojindo, Kumamoto, Japan). After 24 h of treatment with nintedanib, pirfenidone, or D + Q, cells were lysed with 50 μL of lysis buffer and incubated for 10 min. Then, 50 μL of SPiDER-βgal working solution was added to each well and incubated at 37 °C for 30 min. After that, 100 μL of stop solution was added to each well. Fluorescence values were assessed using a fluorescence excitation wavelength of 500 nm and an emission of 540 nm with a fluorescent microplate reader (Biotek, Winooski, VT, USA).

### Cell viability

Cell viability was evaluated using AlamarBlue Cell Viability Reagent (ThermoFisher Scientific). Senescent lung fibroblasts (3 × 10^4^ cells/well) were treated with nintedanib (Ofev, Boehringer Ingelheim, Germany; 300 nM), pirfenidone (Esbriet, Genentech, San Francisco; 2.5 mM) or dasatinib (Tocris, Bristol, UK; 20 μM) + quercetin (Sigma-Aldrich, St. Louis, MO; 15 μM) for 24 h followed by 3 h with the cell death ligand Super Fas Ligand (100 ng/ml). AlamarBlue Cell Viability reagent was added to the cells and incubated for 4 h at 37 °C. Fluorescence values were assessed using a fluorescence excitation wavelength of 560 nm and an emission of 590 nm with a fluorescent microplate reader (Biotek). Results were expressed as fold-change compared to control cells.

### Caspase-3 assay

The effect of SOC drugs on caspase-3 activity was observed by using Caspase-3/CPP32 Fluorometric Assay Kit (BioVision Inc). Senescent fibroblasts were treated with SOC drugs or D + Q for 24 h, followed by 3 h of Super FasL stimuli. Cells were lysed in 50 μl chilled cell lysis buffer on ice for 10 min before 50 μl of 2× reaction buffer (containing 10 mM Dithiothreitol) was added, followed by 50 μM DEVD-AFC substrate, incubated at 37 °C for 2 h. Fluorescence was measured at 505 nm with a fluorescent microplate reader (Biotek). Results were expressed as fold-change compared to control cells.

### Lactate dehydrogenase (LDH) assay

To perform the assay, 50 μL of CyQUANT LDH Cytotoxicity Assay Kit reaction mixture was added to cell supernatants. After 30 min of incubation at RT, protected from light, the assay was stopped with a stop solution. Absorbance was measured at 490 nm and 680 nm using a microplate reader (Biotek, Winooski, VT, USA). Results were expressed as fold-change compared to control cells.

### Soluble collagen-1 direct ELISA

Senescent lung fibroblasts were plated into a 96-well plate and treated with nintedanib, pirfenidone, or D + Q for 24 h. After 24 h, lung fibroblast conditioned supernatants were harvested, and collagen-1 was assessed as previously described^52^. Results were expressed as fold-change compared to control cells.

### Quantitative real-time polymerase chain reaction (qRT-PCR)

Cells were lysed in Trizol™ reagent (Thermo-Fisher Scientific), and RNA was extracted as recommended by the manufacturer. 3 μg of RNA was reverse transcribed into cDNA using SuperScript™ II Reverse Transcriptase (Thermo-Fisher Scientific) as previously described^53^. Gene expression analyses were performed using TaqMan master mix (Thermo-Fisher Scientific) probes for human *Smooth Muscle Actin Alpha 2 (ACTA2), C–C Motif Chemokine Receptor (CCR)10, CDKN1A, CDKN2A, Collagen (COL)1A, COL3A1, (EPH Receptor A3) EPHA3, Fibronectin (FN)1, GDF15, and WNT16 (*all *Thermo-Fisher Scientific*). Quantitative PCR analysis was performed using Viia7 Thermocycler (Thermo-Fisher Scientific). Results were normalized to *RNA18S5* expression and presented as fold-change values compared to control cells by using DataAssist software version 3.01 (Thermo-Fisher Scientific).

### ELISA

IL-6, IL-8, monocyte chemoattractant protein (MCP)-1, GDF-15 were determined in senescent fibroblast supernatant, and WNT16 levels were determined in senescent fibroblast lysates using a standardized sandwich ELISA technique (R&D Systems, Minneapolis, MN, USA), according to manufacturer’s protocol. Results were expressed as fold-change compared to control cells.

### Western blotting

Cells were lysed using RIPA lysis buffer (Thermo-Fisher Scientific) supplemented with Halt protease and phosphatase inhibitor cocktail (Thermo-Fisher Scientific). Protein concentrations were measured by using a Detergent Compatible protein assay (Bio-Rad Laboratories, Inc., Hercules, CA, USA), and the same amount of protein was loaded into a 4–15% NuPAGE Bis–Tris Protein gel. Gels were transferred using an iBlot Dry blotting system onto nitrocellulose membranes (Thermo-Fisher Scientific), and the transferred samples were blocked for 1 h at RT in 5% non-fat-dry-milk in tris-buffered saline (TBS). Primary antibodies used included: Phospho-MLKL (Cat# 916,895, Cell Signaling, Danvers, MA), Mixed Lineage Kinase Domain Like Pseudokinase (MLKL) (Cat #14993S, Cell Signaling), and B cell lymphoma (Bcl)-2 (Cat#Ab182858, Abcam, Cambridge, UK). Images of chemiluminescent bands were acquired using a Bio-Rad Gel documentation system (Bio-Rad Laboratories, Inc.). Membranes were washed in TBS-T, blotted with anti-tubulin antibody (Abcam CAT#Ab6046), and developed similarly. Image Lab Software version 6.0.1 (Bio-Rad Laboratories, Inc.) was used to perform densitometric analysis. Results were expressed as fold-change compared to control cells.

### Statistics

Statistical analyses were performed using GraphPad Prism 9.1.2 (GraphPad Software, San Diego, CA, USA). Data were presented as standard deviation (SD) and evaluated for significance by one-way Analysis of Variance (ANOVA) followed by Tukey’s test. A p value less than 0.05 was considered statistically significant.

### Study approval

This Institutional Review Board at Cedars-Sinai Medical Center approved all experiments with primary human tissue, and informed consent was obtained before inclusion in the studies described herein. All methods were performed in accordance with relevant guideline and regulations.

## Supplementary Information

Supplementary Information.

## Data Availability

The datasets used and analyzed during this study are available from the corresponding author upon reasonable request.
