# Supplementary material for: Standard of care drugs do not modulate activity of senescent primary human lung fibroblasts
Source: Sci Rep. 2023 Mar 4;13:3654. doi: 10.1038/s41598-023-30844-0 (PMC9985617; doi:10.1038/s41598-023-30844-0)
Supplement: Supplementary file 1 — Supplementary Information. [file 41598_2023_30844_MOESM1_ESM.docx]

**Supplementary Material:**

**Western Blot (full gels):**

1. Vehicle
2. Nintedanib
3. Pirfenidone
4. D+Q

Within red rectangle: representative image

Figure 2e (normal): Bcl-2 (26 kDa)


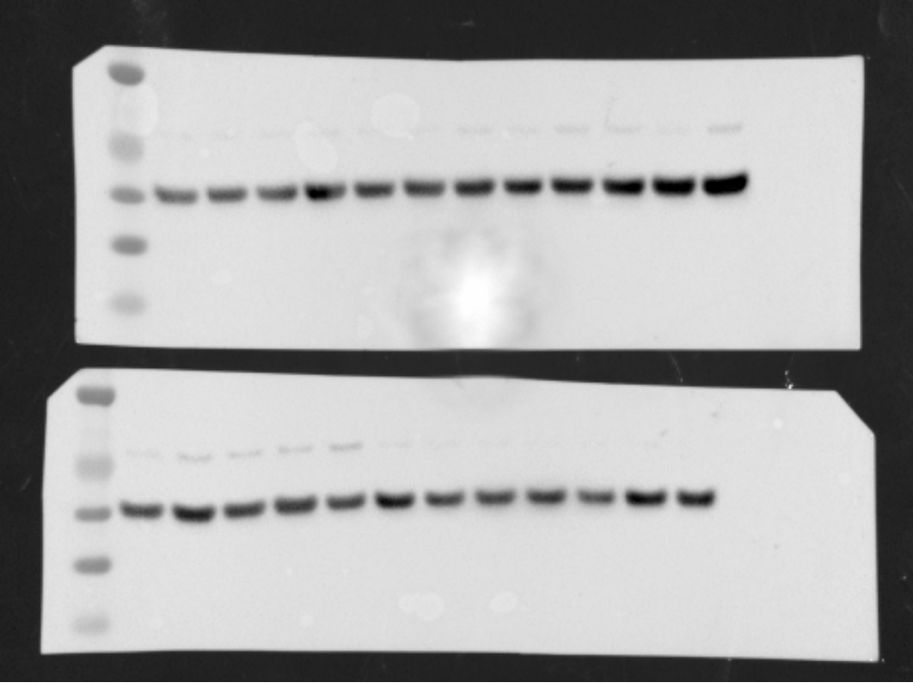


50 kDA

1

2

33

4

1

2

33

4

37 kDA

25 kDA

20 kDA

15 kDA

2

4

33

1

| Lane No. | Total Band Vol. (Int) |
| --- | --- |
| 1 | 4576655 |
| 2 | 4874551 |
| 3 | 4775400 |
| 4 | 7001256 |
| 5 | 6305208 |
| 6 | 6510275 |
| 7 | 6649632 |
| 8 | 6665193 |
| 9 | 7189008 |
| 10 | 7621901 |
| 11 | 8239658 |
| 12 | 9853947 |

Figure 2e (normal): Beta- tubulin (50 kDA)


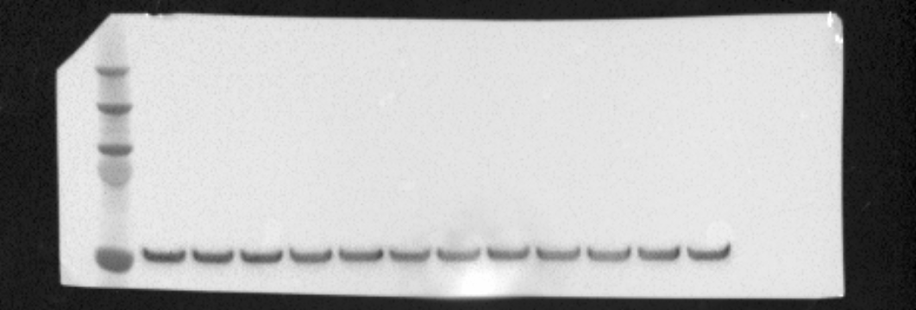


2

33

4

1

4

33

2

1

250 kDA

150 kDA

100 kDA

75 kDA

50 kDA

1

2

33

4

| Lane No. | Total Band Vol. (Int) |
| --- | --- |
| 1 | 4232598 |
| 2 | 3902367 |
| 3 | 4166424 |
| 4 | 3877754 |
| 5 | 4281864 |
| 6 | 4217303 |
| 7 | 2882659 |
| 8 | 3923225 |
| 9 | 4091594 |
| 10 | 3824834 |
| 11 | 4014062 |
| 12 | 4065066 |

Figure 2e (IPF): Bcl-2 (26 kDa)


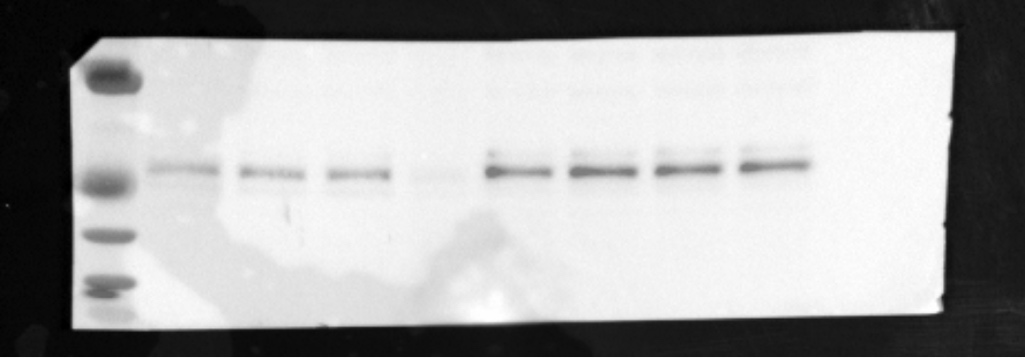


10 kDA

15 kDA

20 kDA

25 kDA

37 kDA

50 kDA

1

2

33

4

4

2

33

1

| Lane No. | Total Band Vol. (Int) |
| --- | --- |
| 1 | 5621595 |
| 2 | 4823784 |
| 3 | 4610026 |
| 4 | 2448659 |
| 5 | 6853380 |
| 6 | 8952064 |
| 7 | 8290870 |
| 8 | 7200792 |

Figure 2e (IPF): Beta- tubulin (50 kDA)


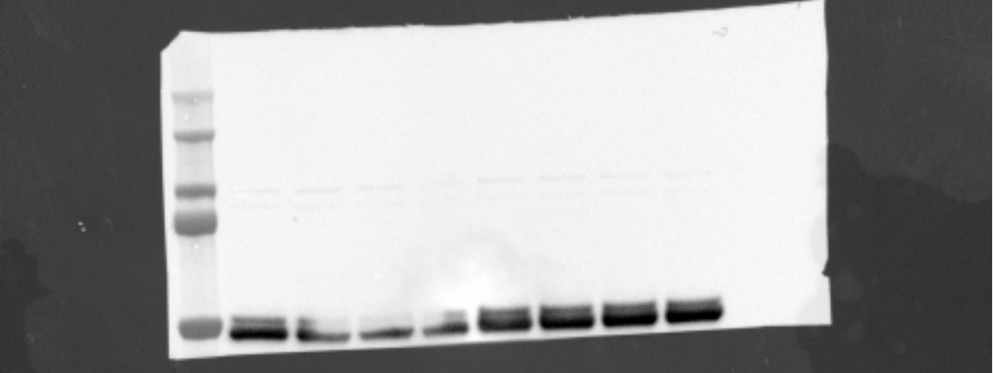


75 kDA

100 kDA

150 kDA

250 kDA

50 kDA

1

2

33

4

4

33

2

1

| Lane No. | Total Band Vol. (Int) |
| --- | --- |
| 1 | 10297900 |
| 2 | 6014190 |
| 3 | 4410700 |
| 4 | 5423496 |
| 5 | 10429911 |
| 6 | 10363275 |
| 7 | 11185300 |
| 8 | 11155430 |

Figure 7b (normal): MLKL total (54 kDa)

250 kDA


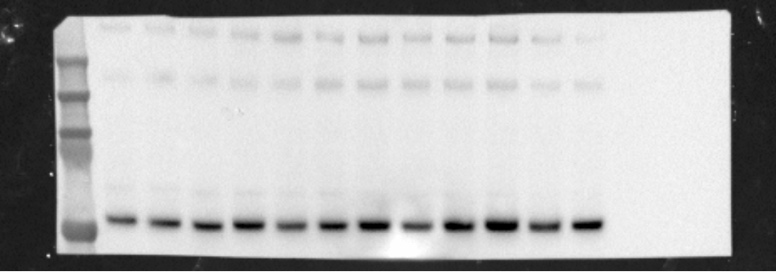


100 kDA

150 kDA

75 kDA

50 kDA

4

33

2

1

4

33

2

1

| Lane No. | Adj. Total Band Vol. (Int) | |
| --- | --- | --- |
| 1 | | 2168628 |
| 2 | | 2624538 |
| 3 | | 2795394 |
| 6 | | 2945649 |
| 7 | | 3695979 |
| 8 | | 2635962 |
| 9 | | 3581340 |
| 12 | | 3543960 |

Figure 7b (normal): phosphorylated MLKL (54 kDa)

75 kDA

50 kDA

250 kDA

100 kDA


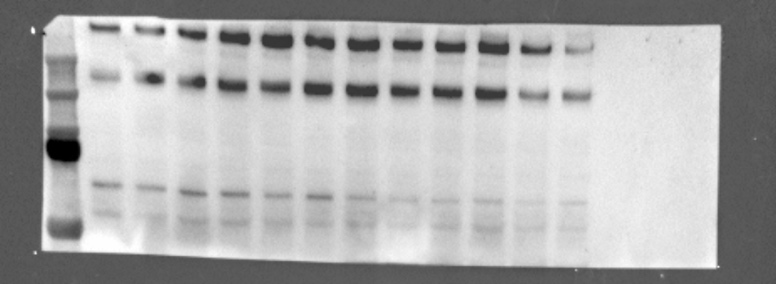


150 kDA

4

33

2

1

4

1

2

33

| Lane No. | Total Band Vol. (Int) |
| --- | --- |
| 1 | 3945627 |
| 2 | 3688839 |
| 3 | 4964757 |
| 4 | 4217598 |
| 5 | 4768449 |
| 6 | 4294353 |
| 7 | 3847599 |
| 8 | 3348450 |
| 9 | 3921666 |
| 10 | 5309892 |
| 11 | 4209093 |
| 12 | 3326127 |

Figure 7b (IPF): MLKL total (54 kDa)


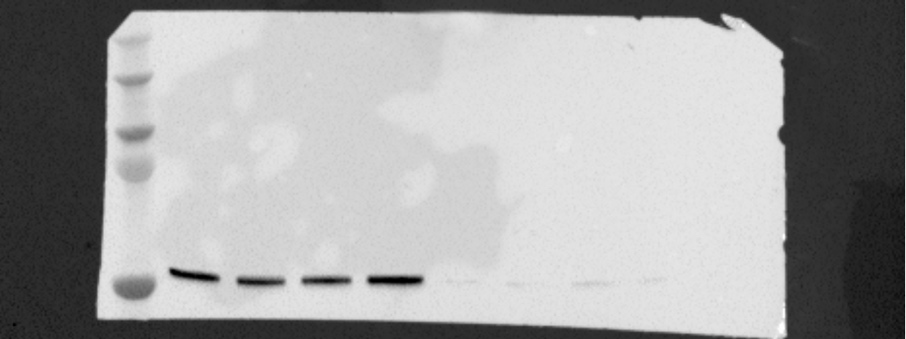


75 kDA

100 kDA

150 kDA

250 kDA

50 kDA

4

33

2

1

4

1

2

33

| Lane No. | Total Band Vol. (Int) |
| --- | --- |
| 1 | 5688870 |
| 2 | 5247284 |
| 3 | 4768456 |
| 4 | 7274464 |
| 5 | 1378748 |
| 6 | 1330560 |
| 7 | 1567076 |
| 8 | 1131452 |

Figure 7b (IPF): phosphorylated MLKL (54 kDa)


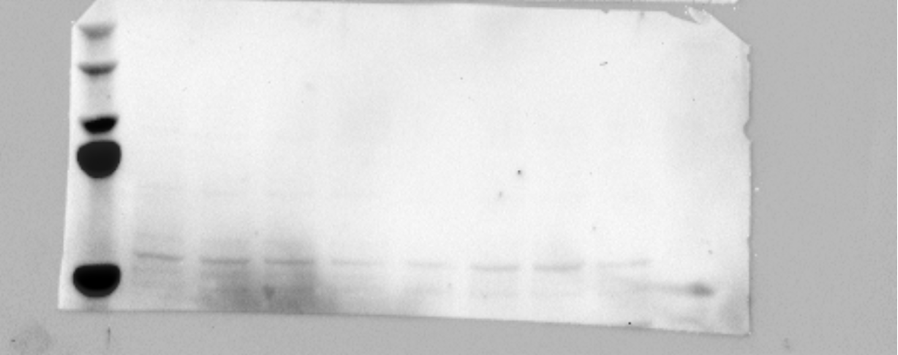


150 kDA

250 kDA

100 kDA

75 kDA

50 kDA

4

33

2

1

4

33

2

1

| Lane No. | Total Band Vol. (Int) |
| --- | --- |
| 1 | 6424628 |
| 2 | 6148492 |
| 3 | 6758892 |
| 4 | 2521456 |
| 5 | 1445164 |
| 6 | 2166696 |
| 7 | 2914184 |
| 8 | 1761228 |
